# BRONCHOLITHIASIS

**DOI:** 10.4103/0970-2113.45280

**Published:** 2008

**Authors:** Sajal De, Sarmishtha De

**Affiliations:** 1 Department of Pulmonary Medicine, Bhopal Memorial Hospital and Research Centre, Bhopal, India; 2 Department of ENT, People's Medical College, Bhopal, India

**Keywords:** Pulmonary Tuberculosis, Broncholith

## Abstract

A 47 year old female who had past history of incomplete treatment for pulmonary tuberculosis presented with increased breathlessness, generalized swelling and loss of ap-petite for last one month. X-ray chest PA view showed bilateral fibrocalcific opacities with blunting of costophrenic angle on both sides. She underwent bronchoscopy to collect bronchial wash to rule out relapse of tuberculosis. On bronchoscopy a loose broncholith with sharp and speculated margins were detected in right middle lobe bronchus. This broncholith was successfully removed through flexible bronchoscope without any complications.

## INTRODUCTION

Broncholiths are calcified material within tracheobronchial tree. They originate from calcified peribronchial lymph nodes which had subsequently erode the bronchus. They may remain asymptomatic or may produce nonspecific symptoms. Clinical signs and radiological investigations are usually inconclusive. Broncholiths are often detected during evaluating symptoms of complications e.g. haemoptysis, recurrent chest infection. Broncholiths should be removed preferably through rigid bronchoscope.

## CASE SUMMARY

A 47 year old female was admitted with complaints of increased breathlessness, generalized swelling of the body and loss of appetite for the past one month. Five years back, she was diagnosed as a case of smear positive pulmonary tuberculosis. She took the treatment for 2 months and left on her own. She denied any other significant past history. Her family history was unremarkable.

On examination, she was conscious and oriented but dyspneic. Her pulse rate was 110/min, respiratory rate was 30/min and blood pressure was 110/80 mm of Hg. No clubbing or cervical lymphadenopathy was detected but JVP was raised. Respiratory system examination showed laboured breathing with bilateral inspiratory crackles and rhonchi. Abdominal examination showed tender hepatomegaly with ascitis. Oxygen on room air was 88%. Her routine investigation were normal.

ECG showed evidence of cor pulmonale. Echocardiography showed severe pulmonary hypertension with dilated right atrium and poorly contracting dilated right ventricle. X-Ray chest (Fig. I) showed mediastinal widening with bilateral fibrocalcific opacity. Costophernic angle on both sides were blunt.

**Fig. 1:**
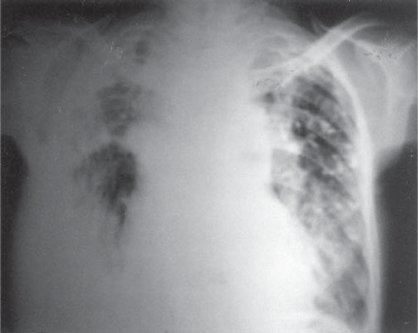
X Ray Chest PA view

USG abdomen showed moderate to gross ascitis with bilateral pleural effusion with normal kidney and altered hepatic echotexture. Diagnostic ascitic and pleural fluid aspiration were done.

Pleural fluid total protein was 0.92g/dl, Sugar108mg/ dl, LDH 110U/L; Total Cell Counts 195/mm^ 3^ (Polymorph 09%, Lymphocyte 88%, Mesothetlial cells 03%). Ascitic fluid shows total protein 1.39 mg/dl, LDH 130U/L, Total Cell Count 160/cm^ 3^ (Lymphocyte 95%, Neutrophil 04%).

CT scan thorax showed large (2x2 cm) calcified right paratracheal lymphadenopathy with multiple bilateral calcified hilar lymph node with bilateral pleural effusion. Fig. II shows calcified right hilar lymph node protruding inside the right middle lobe bronchus.

**Fig. 2:**
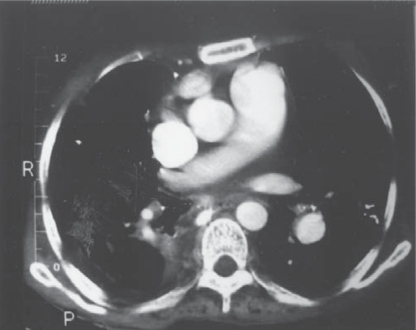
CT thorax showing right hilar lymphadenopathy

As she was not able to produce sputum, she under went videobronchoscopy. On bronchoscopy a large loose broncholith was seen at the opening of right middle lobe bronchus (Fig. III). The margin of broncholith was sharp and speculated. Broncholith was successfully removed by the bronchoscope and no complication was observed. Bronchial aspirate taken from both side of lung which was negative for acid fast bacilli and no organism was grown on culture. Smear prepared with the fragments failed to show any organism.

**Fig. 3:**
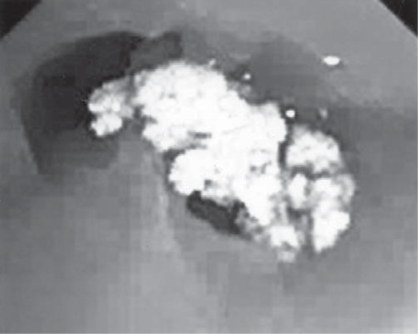
Broncholith in side the right middle lobe bronchus

Subsequently pulmonary function test was done which showed Forced Vital Capacity (FVC) 0.421 (16% of predicted), Forced Expiratory Volume in 1 sec (FEV_1_) was 0.351/s (16% of predicted) and FEV_1_/FVC 65.19% and there was no reversibility. She was unable to perform lung volumes and diffusion. She treated with bronchodilator and diuretic therapy and was discharged with improved condition.

## DISCUSSION

Broncholiths are endogenous calcified material within tracheobronchial tree. According to extended definition^1^, broncholithiasis include all patients with peribronchial calcific node with distortion of the bronchi, as demonstrated by roentgenography or by bronchoscopy.

Broncholiths are classified into two groups^2^ according to their origin: (a) Intrinsic calculi, developed from the lung, bronchi and lymph nodes and which may be subdivided as follows: (i) Senile calcifications\of the elastic cartilage of the bronchi and subsequent sequestration. (ii) Metastatic calcification due to hyperparathyroidism, multiple myeloma, renal rickets, etc. This type of calcification occurs peripherally and rarely results in broncholithiasis. (iii) Dystrophic calcification of necrotic, inflamed or degenerated tissue (the most common mechanism).(b) Extrinsic calculi, developed from aspirated foreign bodies, secretions and dusts (rare).

Broncholithiasis has a various etiology. The commonest etiology is granulomatous lymphadenitis due to fungal or mycobacterial infection. The calcified peribronchial lymph nodes may erode the tracheobronchial tree due to respiratory movement or cardiac pulsation. Preferential sites are known to be the proximal right middle lobe bronchus and the origin of the anterior segmental bronchus of the upper lobes because of the airway anatomy and lymph node distribution.^3^

The broncholiths are usually gray-white and variable in size and usually irregular in shape and often possess spurlike projections or sharp edges. The chemical composition is of calcium phosphate (85-90%) or calcium carbonate (10-15%).

Organisms are rarely detected in extracted broncholiths. However, Weed et al^4^ histopathologically examined broncholith from 9 patients and observed that they had originated from either Histoplasma capsulatum or Nocardial infection. Broncholiths arise within intrathoracic tissues previously infected with tubercle bacilli. However, broncholiths are infrequent during active pulmonary tuberculosis. Stivelman^5^ reported one case in 5,000 active tuberculosis while Zahnlo^6^ noted a single case among 4,000 patients.

Broncholiths can cause distortion, irritation, and erosion of bronchus. Pulmonary signs and symptoms are non specific. Most common presentations^7^ are chronic cough (100%), fever (50%-60%) hemoptysis (45-50%), localized wheezing (25-60%), and chest pain (20%) stone expectoration (15-26%). Rare complication like recurrent pneumonia, massive haemoptysis and fistulas between the bronchi^8^ and adjacent mediastinal structures^9^ had also been reported.

Radiologically broncholiths are very difficult to diagnose. CT findings^10^ of a suspected broncholith is either endobronchial or peribronchial calcified nodule which is associated with findings of bronchial obstruction. However, with conventional CT, it can be difficult to determine whether a calcified nodule is endobronchial in position because thicker sections result in a volume-averaging artifact of broncholith, bronchus, and peribronchial tissues including adjacent lymph nodes. High resolutions CT scan have better ability to identify broticholiths^11^.

Treatment options are simple observation, bronchoscopic removal or surgery. Spontaneous broncholith expectoration may occasionally lead to resolution of symptoms.

Management of the large symptomatic broncholith is controversial. Review of literature^12^ suggested bronchoscopic removals should be considered in cases of uncomplicated and loose broncholithiasis, whereas surgical management should be chosen in complicated cases. The majority of broncholiths can be managed by bronchoscope. Nollet AS et al^13^ suggested broncholiths should be removed under rigid bronchoscope. As broncholiths are usually associated with extensive granulation tissue, removal of symptomatic broncholith by fiber optic bronchoscope is difficult and may cause massive bleeding^14^. Large broncholith can be fragmented by YAG laser and subsequently fragments can be removed by fibre optic bronchoscope^15^. However, safe removal of partially embedded broncholiths by flexible bronchoscopy had been reported^8^.

We are reporting a case of uncomplicated asymptomatic post tubercular broncholith which was successfully removed by the flexible bronchoscope.
